# Cutaneous Pseudolymphomas of the Scalp Secondary to Lamotrigine

**DOI:** 10.7759/cureus.94269

**Published:** 2025-10-10

**Authors:** Juliana M O'Reilly, Ruby Gibson, Jessica Kalen, Pooja Khera

**Affiliations:** 1 Graduate Medical Education, Walter Reed National Military Medical Center, Bethesda, USA; 2 Dermatology, George Washington University School of Medicine and Health Sciences, Washington, DC, USA; 3 Dermatology, Washington DC Veterans Affairs Medical Center, Washington, DC, USA

**Keywords:** cutaneous pseudolymphoma, drug-induced, lamotrigine, mimicker, pseudolymphoma

## Abstract

We present a case of a 25-year-old woman with bipolar disorder that developed five asymptomatic, enlarging scalp nodules over nine months while on a stable dose of lamotrigine. A biopsy revealed mixed B-cell and T-cell lymphocytic infiltrates, and after self-tapering the medication, the lesions shrank. The patient was switched to lurasidone, leading to complete resolution of the scalp nodules. This case supports lamotrigine-induced cutaneous pseudolymphoma, a rare but reversible condition, and emphasizes the need for clinicians to consider drug-induced pseudolymphoma.

## Introduction

Lamotrigine is a commonly prescribed antiepileptic medication used in the management of seizure disorders and bipolar disorder. While dermatologic adverse reactions such as exanthems, erythema multiforme, Stevens-Johnson syndrome, and drug reaction with eosinophilia and systemic symptoms are well recognized, lamotrigine-induced cutaneous pseudolymphoma is rare [1-3]. Cutaneous pseudolymphoma, also known as cutaneous lymphoid hyperplasia, represents a benign lymphocytic infiltrate of the skin that clinically and histologically mimics lymphoma. Triggers include arthropod bites, infections, tattoos, metal implants, allergens, and certain medications [4]. Although lamotrigine-associated pseudolymphoma has been reported in only a handful of cases, recognition is important as the condition is reversible with withdrawal of the offending agent. We present a case of lamotrigine-induced cutaneous pseudolymphoma in a young woman, highlighting the importance of a thorough medication review, including brand-name to generic transitions, when evaluating new skin lesions.

## Case presentation

**Figure 1:**
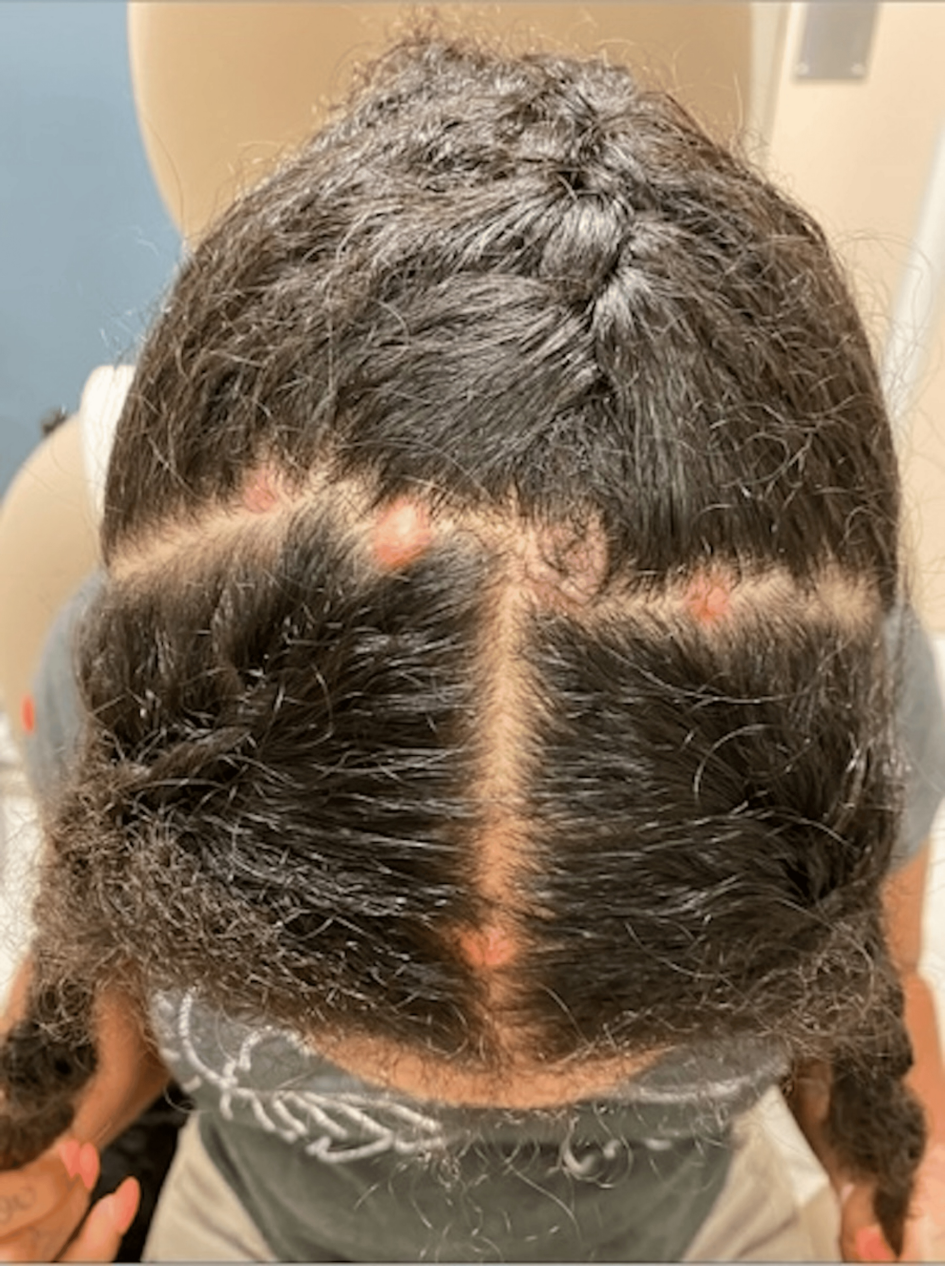
Initial clinical presentation The 25-year-old female patient initial presentation with multiple 1×1cm pink firm nodules on the scalp.

A 25-year-old African American woman with a history of bipolar disorder on lamotrigine presented to the dermatology clinic for scalp lesions. She had five asymptomatic, slowly enlarging smooth nodules on the scalp that started nine months prior to presentation. One lesion was biopsied at an outside hospital that showed atypical lymphoid infiltrates with no evidence of T-cell or B-cell clonality. The patient denied any new medications or changes in dosage of the lamotrigine. She had been on a stable dose of lamotrigine for three years prior to presentation, but she noted switching to a different brand of medication. However, she was unsure of the timeline in relation to the change of brand and the lesions. No constitutional symptoms were present. The patient denied any personal or family history of malignancy. Physical examination revealed a 1 cm pink dome-shaped, soft, shiny nodule with evidence of central hairs on the frontal scalp. The patient had four similar pink nodules on the vertex scalp (Figure 1).

**Figure 2:**
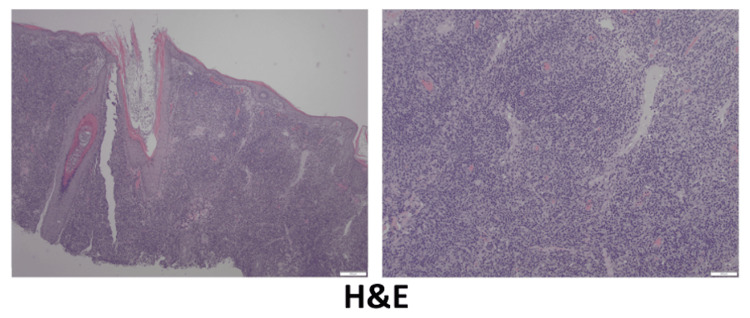
Histology findings Hematoxylin and eosin staining: 40x (left) and 100x (right) showing dense infiltrate of lymphocytes and histiocytes in the dermis.

A repeat biopsy from the frontal scalp nodule was performed, revealing mixed B-cell and T-cell lymphocytic infiltrate. Hematoxylin and eosin (H&E) staining showed dense infiltrate of lymphocytes and histiocytes in the dermis (Figure 2). The lymphocytes were cytologically bland without significant atypia.

**Figure 3:**
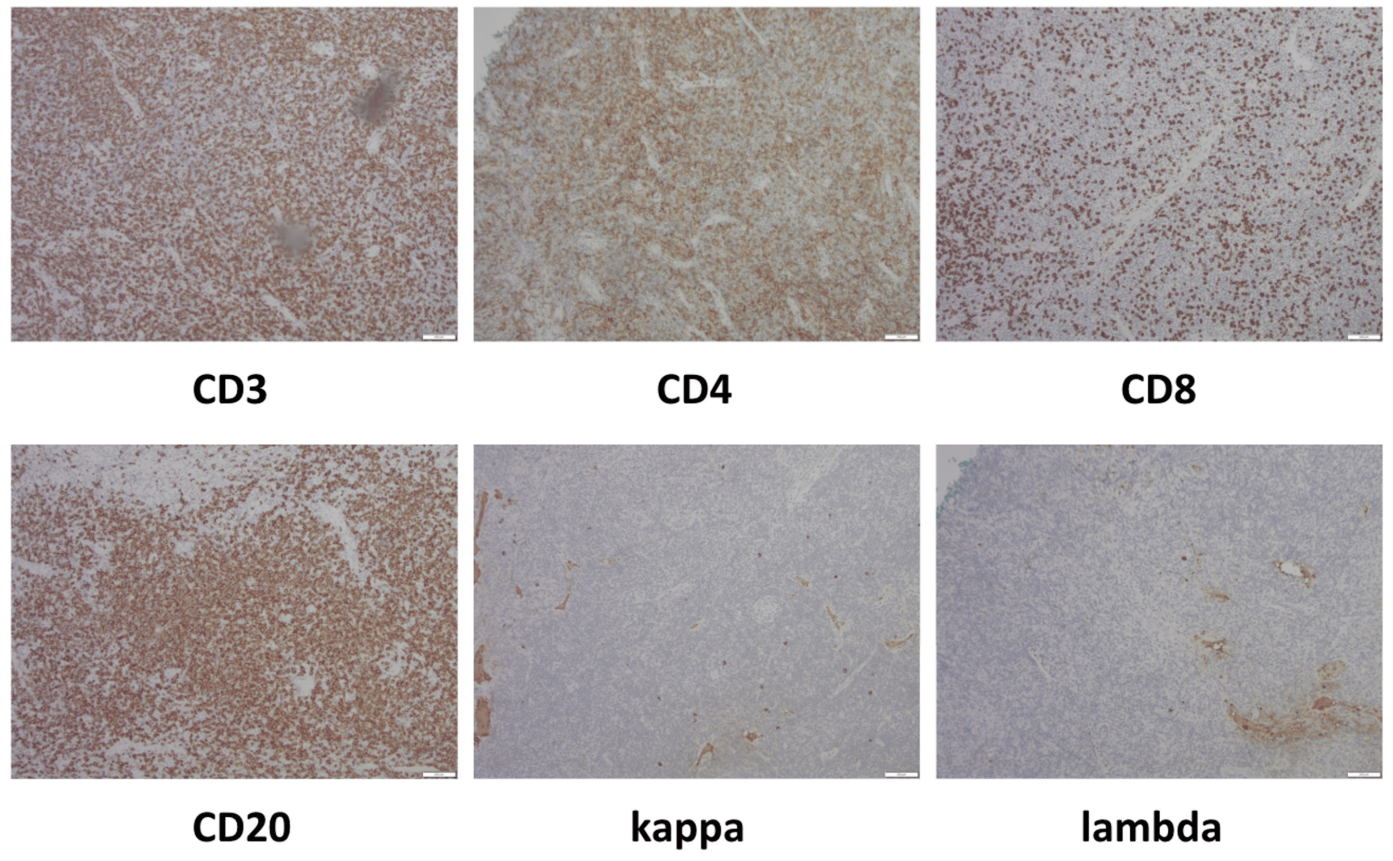
Histology findings Immunohistochemistry of CD3, CD4, CD8, CD20, kappa, and lambda stains at 100x showed a mixed infiltrate of CD20-positive B cells and CD3-positive T cells, with CD4 predominating over CD8. Plasma cells demonstrated polyclonal kappa and lambda light chain expression.

Immunohistochemistry studies demonstrated a mixed population of CD20-positive B-cells and CD3-positive T-cells with a greater distribution of CD4 over CD8. Plasma cells showed a polyclonal expression of kappa and lambda light chains (Figure 3). No immunoglobulin heavy-chain monoclonal rearrangement was detected. T-cell receptor monoclonal rearrangement was indeterminate.

**Figure 4:**
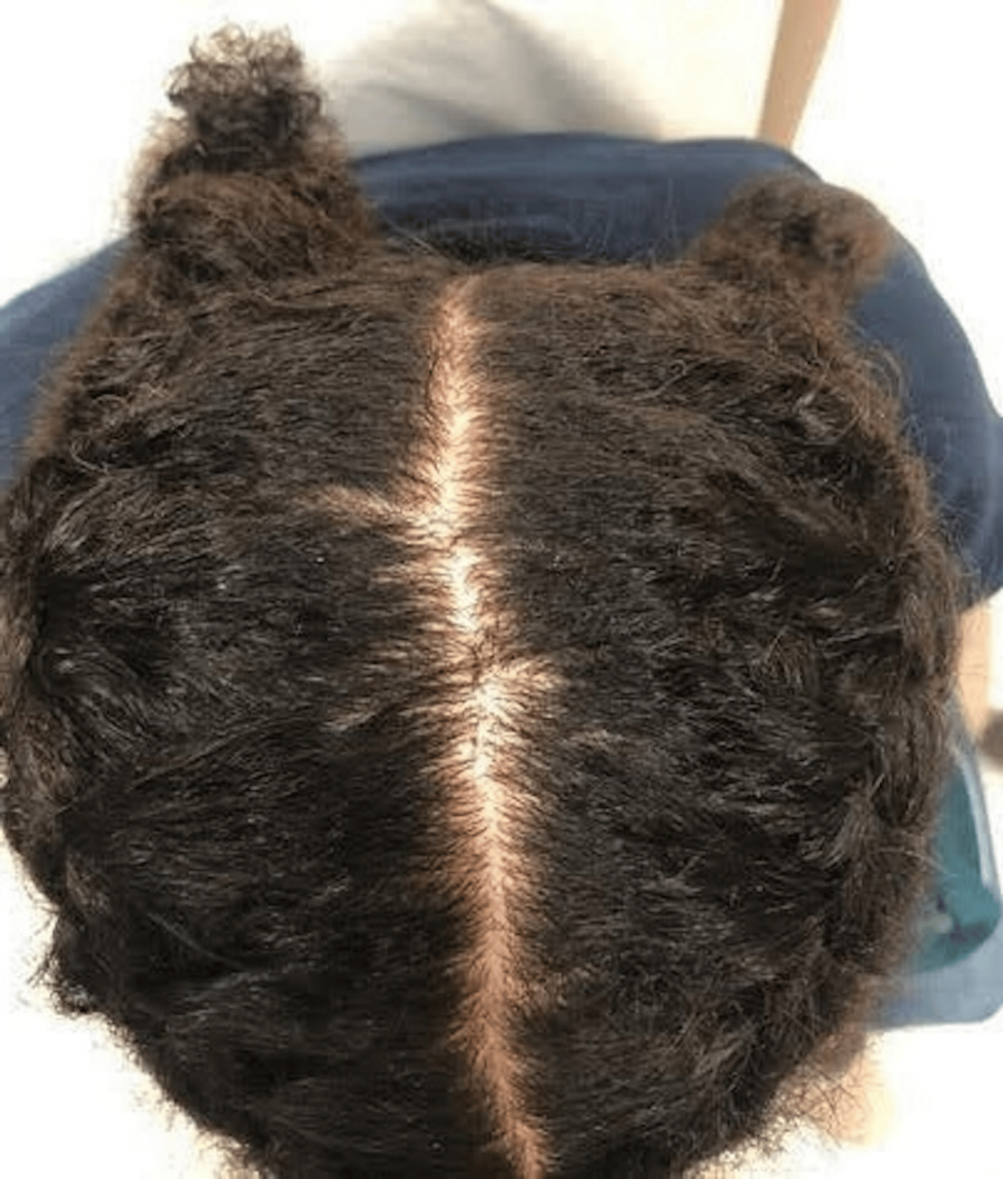
Clinical presentation at follow-up The 25-year-old female patient at one month follow-up after discontinuation of lamotrigine. Nodules had significantly decreased in size compared to the initial presentation.

At one-month follow-up, she had self-tapered to a lower dose of lamotrigine with a subsequent shrinkage of lesions (Figure 4). 

Ultimately, the patient was transitioned to lurasidone by her psychiatrist with complete resolution of nodules. She has remained free of recurrence four years later. The complete resolution after discontinuation of lamotrigine further supports the diagnosis of cutaneous pseudolymphoma secondary to lamotrigine. 

## Discussion

Lamotrigine is an antiepileptic used in the treatment of seizure and psychiatric disorders. Common dermatologic adverse reactions of lamotrigine include erythema, erythema multiforme, exanthems, drug-induced lupus, pruritus, Stevens-Johnson syndrome, toxic epidermal necrolysis, anticonvulsant hypersensitivity syndrome and drug reaction with eosinophilia and systemic symptoms [1-3].

Cutaneous pseudolymphomas, also referred to as cutaneous lymphoid hyperplasia or lymphocytoma cutis, are benign lymphocytic infiltrates of the skin that either clinically and or histologically mimic lymphomas. It is not a single disease but an immunologic reaction to a stimulus including arthropod bites, infections, allergens, tattoos, metal implants, and medications. Clinically, pseudolymphoma presents as isolated, firm, erythematous to violaceous, nodules and plaques, often accompanied by pruritus. Histologic features include reactive germinal centers, polyclonal, and mixed inflammatory dermal infiltrate, including eosinophils and plasma cells [4]. In the majority of cases, particularly medication- or drug-induced pseudolymphomas, resolution has been reported in up to 88.7% of cases following cessation of the offending agent [5].

Lamotrigine-induced cutaneous pseudolymphoma has been reported in a few other cases. One case occurred in an eight-year-old girl who developed slow-growing, painful, pink indurated nodules on the frontal and vertex scalp six months after switching to lamotrigine from oxcarbazepine. Biopsy revealed a dense, nodular, dermal lymphoid infiltrate. Within a few weeks of cessation of lamotrigine, the scalp lesions resolved [6]. Another case was in a 59-year-old woman with a long-standing history of bipolar II, who had multifocal and recurrent cutaneous pseudolymphoma that was found to be associated with lamotrigine [7].

In our patient, the only medication change was from a brand-name to a generic product. To our knowledge, no cases of cutaneous pseudolymphomas occurring solely after a product brand switch have been reported in the literature. While brand changes generally do not alter the active ingredient, they may involve differences in excipients or inactive ingredients, which are not strictly regulated [8]. A limitation of this report is that we did not have access to the patient’s outside medical records to verify the specific brand of her prior medication, and she was unable to recall timing or specific product names. We therefore speculate that an immunologic reaction to an inactive ingredient, potentially through mechanisms that impair immunosurveillance and permit abnormal lymphocyte proliferation, may have contributed to the development of the pseudolymphomas [5]. Importantly, pseudolymphomas can develop across a wide range of time intervals following medication initiation, with a recent review reporting a median onset of 120 days (range 1 to 7,300 days) [5]. Thus, it cannot be determined with certainty whether the brand-to-generic switch directly triggered this immunologic response; however, it remains an important factor to consider.

We hope this case encourages clinicians to consider lamotrigine as a potential drug-induced cause of cutaneous pseudolymphoma, regardless of the time elapsed since the initiation of treatment. Additionally, we urge clinicians to review not only medications and dosing changes but also any transitions from brand-name to generic drugs. Given the recognized low risk of malignant transformation associated with cutaneous pseudolymphoma, we also recommend continued follow-up for these patients [7].

## Conclusions

The temporal association between medication exposure and disease development in this case supports a diagnosis of lamotrigine-induced cutaneous pseudolymphoma, a rare but reversible adverse drug reaction. This condition underscores the importance of a thorough medication review - including consideration of brand-to-generic switches - when evaluating patients with new pseudolymphomatous eruptions. Because these lesions can closely mimic cutaneous lymphoma, accurate diagnosis relies on careful clinicopathologic correlation to avoid unnecessary anxiety, invasive procedures, or inappropriate treatment.
